# Malunion of the distal radius in children: accurate prediction of the expected remodeling

**DOI:** 10.1007/s11832-016-0741-9

**Published:** 2016-05-20

**Authors:** J. A. van der Sluijs, J. L. Bron

**Affiliations:** Department of Orthopaedic Surgery, VU University Medical Center, Amsterdam, The Netherlands

**Keywords:** Malunion, Remodeling, Distal radius fractures, Prediction

## Abstract

**Purpose:**

Malunions of fractures in children have a natural tendency to remodel. However, quantitative data of this well-known process are scarce. The extent of the correction depends inter alia on the type of bone and the location of the deformity and growth remaining. The aim of this study was to quantify the remodeling process of distal radius malunions in children to allow better future prediction.

**Methods:**

Data were derived from two published patient series. Analysis included 63 malunions of distal radius fractures in 62 children (38 boys), with a mean age of 8.5 years (range 2–14.5 years).

**Results:**

The mean initial dorsovolar angulation was 25º [standard deviation (SD) 7.8°], remodeling time 22 (SD 18) months, and angulation at follow-up 6.7° (SD 5.8°). Based on these findings, the remodeling process can be described as an exponential function with angulation (*A*_0_) as a factor and the remodeling time (RT) as a negative exponent of *e* (*R*^2^ = 0.47). The function allows accurate prediction of the expected correction in over 76 % of the malunions. From this model, a formula was derived for calculation of the time needed for complete remodeling, but this formula lacked precision when compared to findings in the literature and needs to be validated.

**Conclusions:**

The remodeling of distal radius malunions can be described as an exponential function with starting speed dependent on the initial angulation. The current model proves to be more accurate than models described previously in the literature. These findings allow for better patient information and optimal planning of eventual surgical intervention. The postulated model could serve as a basis for the description of correction of other malunions by adaptation of the coefficients in this model.

## Introduction

Distal radius fractures are the most common fractures occurring in childhood and a substantial proportion of these patients will develop malunions initially. Fortunately, malunions in children often show a tremendous remodeling potential and initial treatment can usually be restricted to the reassurance of the parents of the involved child. However, although this is a well-known practice for most doctors treating children with fractures, surprisingly few studies (*n* = 7) are available with quantitative data on the dynamics of remodeling. The time needed for the remodeling process is unknown, which impedes the prediction of outcome and, thus, proper patient information. Reported remodeling times (RT) to full correction vary between a mean of 4 months [1–3] and 5 years [4] in the literature. In addition, the speed of remodeling has been shown to vary between 0.9° to 2.5°/month [5–7]. Greater angulated fractures tend to remodel at a faster rate [5, 7]. Hence, the use of a general remodeling speed to predict RT to full correction is not feasible. Friberg, therefore, developed a (exponential) model using the primary malunion angulation (*A*_0_) to describe the residual angulation (*A*_T_) in distal radius malunions [5]. The model, however, lacks accuracy and is, therefore, only rarely used in orthopedic practice.

The aim of the present study is to develop a model which accurately predicts the dynamics of the remodeling process. We use the remodeling data of two previously published studies to modify Friberg’s model in order to enhance its accuracy. In addition, we develop a model to calculate the time needed for complete remodeling. These models should allow to provide a more evidence-based patient education and select those malunions that will not sufficiently remodel and require intervention.

## Patients and methods

We used data from two published cohorts of children with distal radius fractures with dorsovolar angulation. Cohort A is from a study on the remodeling of malunions of forearm fractures which presents a table with patient data on 36 children [4]. From this table, were selected the malunions in the distal third of the forearm in dorsovolar dislocation (*n* = 31). Cohort B was derived from a study on the remodeling speed of distal radius fractures with dorsovolar angulation more than 15° (*n* = 32) [7]. Angle measurements in both cohorts were identical: the central longitudinal intramedullary axis was determined in both the proximal and (angulated) distal fragment. The angle between these two axes was used as the angulation angle. This method was described by Hansen et al. [8].

From all the included patients, we assessed age at time of fracture, gender, malunion angulation (*A*_0_) in the dorsovolar direction, angulation at follow-up, and time of follow-up (= RT). Because both studies were retrospective, the follow-up times (= RT) differ. The difference between initial malunion angulation (*A*_0_) and angulation at follow-up (*A*_T_) was defined as remodeling, measured in degrees.

Using the data from the combined cohort, two models were evaluated: Firstly, a prediction model was formulated based on the findings by Friberg [5]: $A_{T} = A_{0} \times e^{ - C \times RT}$ and, secondly, we modified this model with a second coefficient to study the influence of *A*_0_: $A_{T} = B \times A_{0} \times e^{ - C \times RT}$ (the coefficients were calculated using the nonlinear regression function of SPSS, see below).

### Statistical analysis

All data were analyzed using SPSS (version 15.0, SPSS Inc., Chicago, IL, USA). The results are presented as means (standard deviation, SD). Nonlinear regression was used to estimate the coefficients of the models. For the Friberg-based model, we started with the coefficient found in that study. For the modified model, the starting value for the second coefficient was the value found in the study of Jeroense et al. [7]. The significance of the difference of the parameters and differences between subgroups was tested using the *t* test. To test the precision of the prediction of the models, we compared predicted and observed RT using parametric techniques (*t* test). The best of the two models was subsequently used to estimate time needed to complete remodeling. All tests are two-tailed and considered significant if *p* < 0.05.

## Results

Data are based on the analysis of 63 dorsovolar malunions of the distal radius: 31 from the study by Gandhi (A) (cases 1–31 in the patient data table) and 31 patients (32 malunions) from the study by Jeroense (B) (see Appendix). There were 38 boys, with a mean age of 8.5 years (range 2–14.5 years). The mean malunion angulation was 25° (SD 7.8), mean remodeling time 22 (SD 18) months, and mean angulation at follow-up 6.7° (SD 5.8). The cohorts showed differences in follow-up time (35 vs. 9 months) and final angulation (see Table 1).

**Table 1:** Summary of the data from 62 patients

	Gandhi cohort (A), *N* = 31	Jeroense cohort (B), *N* = 31	Difference	Significance
Age (years)	7.7	9.1	1.3 years	0.043
Remodeling time (months)	35	9	25 months	0.000
Malunion angulation (*A*_0_)	26°	24°	2.5°	0.1
Angulation at FU	5°	8°	3.5°	0.02

Comparison of the two subgroups

### Prediction of remodeling

#### Friberg’s exponential model

Using Friberg’s model for the combined cohort, the prediction coefficient was 0.13 [confidence interval (CI): 0.1–0.16), with a low precision (*R*^2^ = 0.11). Using the model for subgroup analysis (cohorts A and B), we found significant differences in the coefficient of remodeling with B coefficients of 0.06 (95 % CI: 0.068–0.045) and 0.17 (95 % CI: 0.21–0.13), respectively (*p* < 0.05).

#### Modified exponential model

We developed a modified model by adding a second coefficient to modify A_0_. The best fit is the model $A_{T} = 0.51 \times A_{0} \times e^{ - 0.034 \times RT}$ (51 % of the starting angulation and a coefficient of 0.034 for RT). This improves prediction for the combined cohort: *R*^2^ = 0.47. With this model, the subgroups did not differ (Table 2). Adding age or gender did not improve the model. Analysis excluding the four patients older than 12 years of age only marginally influenced the results of this nonlinear regression.

**Table 2:** Models of observed remodeling (*A*_T_) and initial malunion angle (*A*_0_) and RT (*n* = 63 malunions)

Model	Dependent variable	Independent variables	Model	95 % CI of coefficient of RT	*R* ^2^
Model of Friberg	A_T_	RT, *A*_0_	$A_{T} = A_{0} \times e^{ - 0.13 \times RT}$	0.1–0.16	0.1
Modified model	A_T_	RT, *A*_0_	$A_{T} = 0.5 \times A_{0} \times e^{ - 0.034 \times RT}$	0.024–0.044	0.47

*A*_T_ remodeling angle, *RT* remodeling time, *A*_0_ initial malunion angulation

### Precision of the exponential models

In both models, the predicted values of remodeling were not significantly different from the observed values. The mean difference between the observed and predicted remodeling based on the Friberg model with the present coefficient was 1.1°. The modified model had a mean difference of 0.07° with the observed values (see Table 3).

**Table 3:** Differences between the observed and predicted remodeling of malunions angulations (*n* = 63)

	Observed remodeling (mean)	Predicted remodeling (mean)	Difference (*p*)	95 % CI of difference	Number of predictions <5°
Friberg model	6.7°	5.6°	1.1° (*p* = 0.1)	−0.2 to 2.5	41/63
Modified model	6.7°	6.6°	0.07° (*p* = 0.89)	−0.9 to 1.1	48/63

**Fig. 1:**
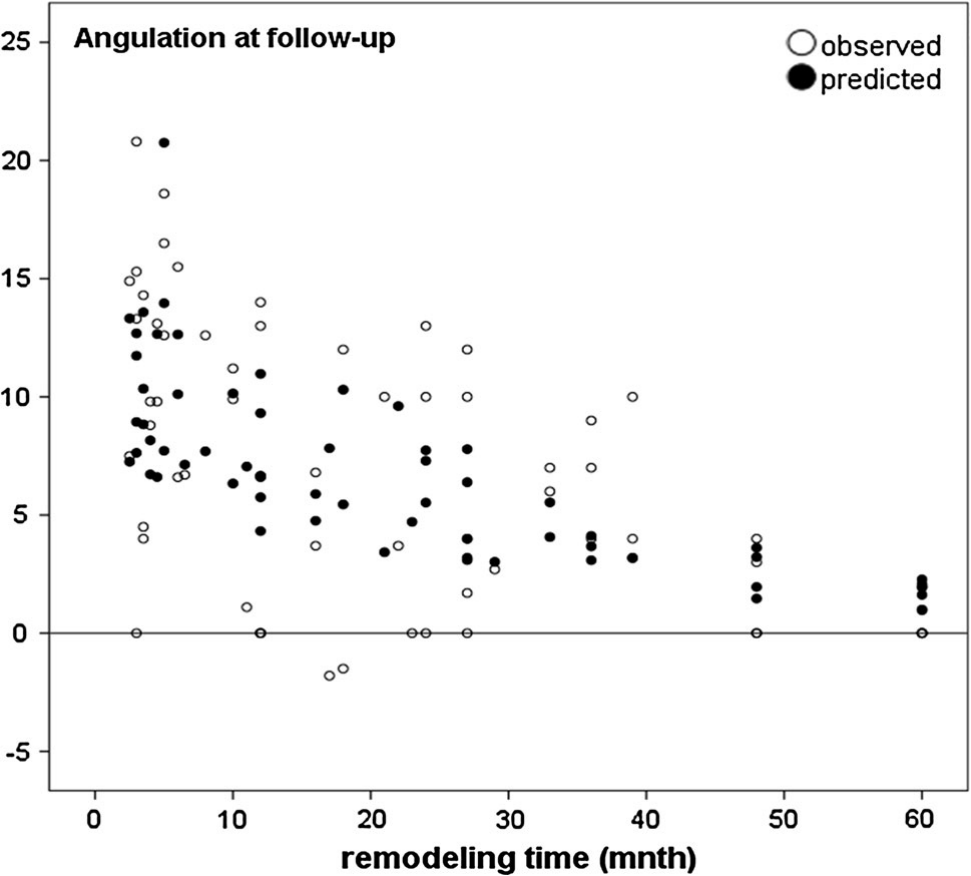
Observed and predicted angulation at follow-up (*A*_T_) of distal radius malunions. On the horizontal axis is the remodeling time, and on the vertical axis, the observed remodeling angulations (°) are shown next to the predicted angulation (*filled circles*) based on the model $A_{T} = 0.5 \times A_{0} \times e^{ - 0.034 \times RT}$

Although the mean differences between predicted and observed values of the original Friberg model was small, the SD was substantial. Using the Friberg model, the values in 41/63 fractures were within 5° of predicted values and, in four cases, differed by more than 10°. Using the modified model, the mean difference was 0.07° (SD 4.2°) and with a smaller SD; 48/63 were within 5° (see Fig. 1).

### Time needed for remodeling

The modified model was used to derive a formula for remodeling time. However, since remodeling is an asymptotic function, completed remodeling cannot be determined with the model. For practical purposes, the value of 3° was considered as adequate remodeling. With *A*_T_ = 3°, derivation from the modified model yields the formula $= \frac{{{ \ln }\left( {\frac{{A_{0} }}{6}} \right)}}{C}$ (see Appendix for derivation). This formula was used to calculate predicted remodeling times with different coefficients in the modified model using values based on the assessed CI (low–mean–high). The mean and low coefficients resulted in RT longer than described in the literature; only the high coefficient yielded values in accordance with the published results. Using the information on remodeling time described in the literature, this study presents an estimated guess of RT depending on malunions angulation in Table 4.

**Table 4:** Estimates of remodeling time of distal radius malunions based on the modified model $RT = \frac{{{ \ln }\left( {\frac{{A_{0} }}{6}} \right)}}{C}$ with fast coefficient *C* and on data from the literature

Malunion angulation	Expected remodeling time, mean (range), months	Based on
15°	12 (2.5–13)	Do study
30°	36 (30–48)	Based on modified model using fast coefficient. Confirmed by Johari and Roth study
40°	40–50	Based on modified model using fast coefficient. Confirmed by Gandhi study

## Discussion

This study shows that remodeling of distal radius fractures can be described as an exponential function. The use of the original model of Friberg turned out to be less accurate, with a low *R*^2^ and only 41/63 (65 %) of the malunions showed final remodeling within 5°. Whereas in the original study the exponential coefficient was 0.087 [standard error (SE) 0.058], the present study found a coefficient of 0.13 (CI: 0.1–0.16). In addition, the two subgroups (cohorts A and B), when calculated according to the Friberg model, showed statistically different values: the oldest (Study A, UK, 1962) has the slowest remodeling (*B* = 0.057), while the most recent (Study B, The Netherlands, 2015) has the fastest (*B* = 0.16).

Using the modified model resulted in a more accurate prediction of the remodeling process, with 48/63 (76 %) malunions within 5°, with an *R*^2^ of 0.45. Moreover, when using this modified model, no differences were found between the two subgroups. The exponential model is better than a linear model but intuitively difficult. For practical purposes, a table has been presented with estimates which can be used for prediction. As a rule of the thumb, the estimated time for remodeling would be around 1°/month for distal radius fractures, with 1.5° in the first 6 months.

Since the modified model proved to be the most accurate predictor of remodeling, this model was used to derive a formula for the remodeling time. However, we found a discrepancy between the remodeling times calculated with our formula for the mean coefficient compared to earlier studies in the literature. For 15° of malunion, the RT estimates would be between 12 and 38 months, which does not agree with the study of Do et al. [1], who showed that angulations below 15° correct spontaneously after an average time of 4 months (range 2.5–13 months); apparently, observed remodeling in the literature is faster in the first year than in the presented cohort. The RT calculation using the high coefficient is the best approximation of the literature. Estimates for that value are still longer than the time reported by Johari [2] (36 months, range 30–48) but agree with Roth et al. [3], who reported 42 months. Moreover, Gandhi’s statement that 95 % of the fractures are corrected after 60 months is correct but might be too conservative.

A limitation of this study is that the distal radius fractures studied are a heterogeneous group with some located proximally in the distal third and some distal in that segment. Since the more proximal fractures remodel slower, this may have caused some of the variability found. In addition, the two cohorts have different follow-up times. This has the advantage of having data with a longer time interval for study but, possibly, differences in the early months are less clearly visible. They are from different decades but that should not affect the underlying biological process. Using the original Friberg model, there seems to be a difference in remodeling behavior, but using the modified model, the differences disappeared. Whether this model only describes the study data or can be generalized remains to be tested.

A final limitation is that the exponential model is asymptotic and never predicts full remodeling. This suggests that corrective growth is not only longitudinal but also shows a tendency to realign to the anatomical axis. For this, the model might be further expanded.

In conclusion, the remodeling process of distal radius malunions in children can be described as an exponential function, with its starting speed dependent on the initial angulation. The current modified model proves to be more accurate than the model derived from the findings of Friberg. In addition, a formula for the prediction of remodeling time, based on the modified model, was described. These models add to our insight of the remodeling process and allow for more evidence-based patient information and optimal planning of eventual surgical intervention. Furthermore, the postulated model could serve as a basis for the description of the correction of other malunions by adaptation of the coefficients in this model.

## Appendix

Derivation of RT using the modified model.

$$A_{T} = 0.5 \times A_{0} \times e^{ - C \times RT}$$

For remodeling to 3°:

$$3 = 0.5 \times A_{0} \times e^{ - C \times RT}$$

$$1 = \frac{{A_{0} }}{6} \times e^{ - C \times RT}$$

$$ln1 = \ln \left( {\frac{{A_{0} }}{6}} \right) - C \times RT$$

$$RT = \frac{{\ln \left( {\frac{{A_{0} }}{6}} \right)}}{C}$$

**Table 5:** Patient data overview

Patients	Case	Age (years)	Sex	Malunion angulation (°)	FU angulation (°)	RT (months)
Cohort A	1	9	M	32	1	60
	2	11	F	30	0	60
	3	4	F	15	0	60
	4	7	M	35	0	60
	5	2	M	25	0	60
	6	8	M	30	0	60
	7	9	M	15	0	48
	8	5	M	37	3	48
	9	4	F	20	4	48
	10	9	M	33	0	48
	11	11	M	24	10	39
	12	7	M	21	4	36
	13	5	M	24	4	39
	14	8	M	28	9	36
	15	9	M	25	7	36
	16	8	M	25	7	33
	17	4	M	34	6	33
	18	12	M	39	12	27
	19	8	M	32	10	27
	20	10	F	20	4	27
	21	8	M	16	0	27
	22	8	M	25	0	24
	23	10	M	33	13	24
	24	9	F	35	10	24
	25	9	M	14	10	21
	26	5	F	38	12	18
	27	7	M	33	13	12
	28	9	M	28	14	12
	29	7	M	20	0	12
	30	7	M	13	0	12
	31	11	M	20	0	12
Cohort B	32	5.5	F	18	10	10
	33	7	F	20	4	16
	34	6.5	M	20	–1.5	18
	35	9	F	15	9	4
	36	7	M	18	7	6.5
	37	5.5	M	29	11	10
	38	3	F	19	10	4
	39	11	F	31	16	6
	40	10.5	M	28	21	3
	41	8	F	25	7	6
	42	8.5	M	26	15	3
	43	8	M	30	13	4.5
	44a	8	F	20	5	3.5
	44b	“	F	17	0	3
	45	8	M	29	15	2.5
	46	4	F	49	19	5
	47	10	F	18	17	5
	48	10	F	16	3	29
	49	10	F	17	7	12
	50	10	F	41	4	22
	51	10.5	F	20	13	3
	52	9.5	M	23	14	3.5
	53	9	F	33	13	5
	54	11	M	31	4	3.5
	55	11	F	16	2	27
	56	7.5	F	16	7	16
	57	12.5	M	21	0	23
	58	12.5	M	20	13	8
	59	14	M	15	10	4.5
	60	11	M	28	–2	17
	61	11.5	F	21	1	11
	62	14.5	M	16	8	2.5

*FU* follow-up, *RT* remodeling time
